# A Sustainable Dual Cross-Linked Cellulose Hydrogel Electrolyte for High-Performance Zinc-Metal Batteries

**DOI:** 10.1007/s40820-024-01329-0

**Published:** 2024-02-02

**Authors:** Haodong Zhang, Xiaotang Gan, Yuyang Yan, Jinping Zhou

**Affiliations:** 1 Hubei Engineering Center of Natural Polymers-Based Medical Materials, Key Laboratory of Biomedical Polymers of Ministry of Education, College of Chemistry and Molecular Sciences, Wuhan University, Wuhan, 430072 People’s Republic of China; 2 Zhongnan Hospital of Wuhan University, Institute of Hepatobiliary Diseases of Wuhan University, Transplant Center of Wuhan University, Wuhan, 430072 People’s Republic of China

**Keywords:** Cellulose, Dual cross-linked, Aqueous rechargeable Zn-metal batteries, Hydrogel electrolyte

## Abstract

Aqueous rechargeable Zn-metal batteries (ARZBs) are considered one of the most promising candidates for grid-scale energy storage. However, their widespread commercial application is largely plagued by three major challenges: The uncontrollable Zn dendrites, notorious parasitic side reactions, and sluggish Zn^2+^ ion transfer. To address these issues, we design a sustainable dual cross-linked cellulose hydrogel electrolyte, which has excellent mechanical strength to inhibit dendrite formation, high Zn^2+^ ions binding capacity to suppress side reaction, and abundant porous structure to facilitate Zn^2+^ ions migration. Consequently, the Zn||Zn cell with the hydrogel electrolyte can cycle stably for more than 400 h under a high current density of 10 mA cm^−2^. Moreover, the hydrogel electrolyte also enables the Zn||polyaniline cell to achieve high-rate and long-term cycling performance (> 2000 cycles at 2000 mA g^−1^). Remarkably, the hydrogel electrolyte is easily accessible and biodegradable, making the ARZBs attractive in terms of scalability and sustainability.

**Supplementary Information:**

The online version contains supplementary material available at 10.1007/s40820-024-01329-0.

## Highlights

- A sustainable dual cross-linked cellulose hydrogel with excellent mechanical strength was fabricated from aqueous alkali hydroxide/urea solution using a sequential chemical and physical cross-linking strategy.
- The hydrogel electrolyte effectively suppresses dendrites growth and side reactions to achieve a stable Zn anode (over 2000 h for Zn||Zn cell), which are proved by a multi-perspective and in-depth mechanism investigation.
- The hydrogel electrolyte is easily accessible and biodegradable, making the zinc batteries attractive in terms of scalability and sustainability.

**Supplementary Information:**

The online version contains supplementary material available at 10.1007/s40820-024-01329-0.

## Graphical abstract

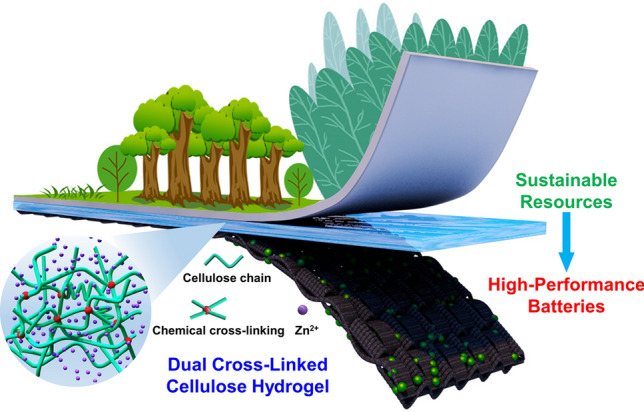

## Introduction

The development of sustainable rechargeable batteries for a high-performance electrical energy storage systems is urgently needed to achieve carbon peaking and carbon neutrality targets [1–3]. Aqueous rechargeable Zn-metal batteries (ARZBs) have garnered widespread interest in recent years on account of their inherent safety, appropriate redox potential (− 0.76 V vs. standard hydrogen electrode), and low cost [4–6]. However, ARZBs face severe challenges related to the Zn-metal anode [e.g., dendrite formation, Zn corrosion, and hydrogen evolution reaction (HER)] during the plating/stripping process, which cause poor reversibility and even short-circuited failure [7]. Optimizing electrolyte composition has been demonstrated as an effective strategy to overcome these obstacles [8], such as electrolyte additive [9], water-in-salt electrolyte [10], eutectic electrolyte [11], and hydrogel electrolyte [12]. Among them, the soft and wet hydrogel electrolyte with rich nanochannels and polar groups can facilitate transport of charge carriers and enhance uniformity of the electric field and Zn^2+^ ions distribution [13]. In particular, the polysaccharide-based hydrogel electrolyte has great potential for application in ARZBs with inherent biocompatibility, biodegradability, and non-toxicity properties [14].

The most investigated polysaccharide-based hydrogel electrolytes are generally based on cellulose [15], chitosan [16], alginate [17], and agarose [18] etc. Thereinto, cellulose is the most abundant natural polysaccharide, composed of d-glucose units linked by *β*-1,4-glycosidic bonds. Cellulose-based hydrogels possess multiple advantages, including abundant hydroxyl (–OH) groups, adequate porosity, and excellent hydrophilicity [19]. These characteristics endow them with a certain mechanical strength that inhibits dendrite formation at the Zn metal/electrolyte interface. Additionally, their unique chemical structure helps alleviate side reactions by limiting the free water content [20]. Nevertheless, their poor mechanical properties cannot meet the requirements for practical applications. So far, many cellulose-based hydrogel electrolytes incorporate kinds of synthetic polymer [e.g., polyacrylamide and zwitterionic poly(sulfobetaine)] to improve the mechanical strength [21, 22]. Even though the mechanical strength has been enhanced to some extent, it leads to the loss of biodegradability and even rise of processing costs. Accordingly, developing a sustainable cellulose-based hydrogel electrolyte that can simultaneously fulfill the mechanical property and cycling stability needed for high-performance ARZBs remains a major challenge.

In this work, we design a dual cross-linked (DC) cellulose hydrogel electrolyte with zinc trifluoromethylsulfonate [Zn(OTf)_2_] salt, denoted by DCZ-gel, which is fabricated from an aqueous cellulose/alkali hydroxide/urea solution using a sequential chemical and physical cross-linking strategy. The unique DC network endows the hydrogel electrolyte with excellent mechanical strength (2.08 MPa, 145%) and abundant porous network for ion transport of 38.6 mS cm^−1^. The interactions between Zn^2+^ ions and –OH groups on cellulose chains could modulate the solvation structure of [Zn(H_2_O)_6_]^2+^. Due to the synergistic effects, the DCZ-gel electrolyte effectively suppresses dendrites growth and side reactions to achieve a stable Zn anode, which was proved by comprehensive experimental results and theoretical calculations. Therefore, the assembled cells [(i.e., Zn||Zn, Zn||Cu, and Zn||polyaniline (PANI)] with the DCZ-gel electrolyte exhibit more excellent electrochemical performances than that with the liquid electrolyte. Moreover, the DCZ-gel electrolyte is characterized by simple processing, low cost, and environmental friendliness, facilitating the development of polysaccharide-based hydrogel electrolyte for energy-storage applications.

## Experimental Section

### Materials

The cellulose sample (cotton linter pulp, over 95% α-cellulose content) was provided by Hubei Golden Ring Co., Ltd (Xiangyang, China), and the viscosity-average molecular weight was measured to be 9.5 × 10^4^ g mol^–1^ (by using a viscometer in cadoxen at 25 °C). Zn(OTf)_2_ was purchased from Macklin Chemistry Co. Ltd. (Shanghai, China). LiOH·H_2_O, concentrated HCl aqueous solution, urea, epichlorohydrin (ECH), ethanol, ammonium persulfate (APS) and aniline were purchased from Sinopharm Chemical Reagent Co. Ltd. (Shanghai, China), and were used without further purification. Deionized (DI) water was used in all the experiments.

### Preparation of Cel-gel, DCH-gel, and DCZ-gel

Cellulose (12 g) was dissolved in 8 wt% LiOH⋅H_2_O/15 wt% urea aqueous solution and then precooled to − 12 °C to form a 6 wt% transparent solution according to our previous method [23]. Then, a certain amount of ECH (i.e., 2, 3, 4, or 5 mL) was added dropwise into the stirring cellulose solution. After the air bubbles were removed by centrifugation, the transparent and viscous cellulose solution containing ECH was spread onto a 1 mm thick glass plate or poured into a 24-well plate (1.5 cm in diameter and 1 cm in height). Then, it was maintained at 5 °C for 24 h for the chemical cross-linking reaction between the –OH groups on the cellulose chains and the chlorine and epoxy groups of ECH. The obtained cellulose hydrogels were then removed from the mold and immersed in 75% (v/v%) aqueous ethanol solution at 5 °C for 6 h to terminate the chemical cross-linking reaction and simultaneously induce physical cross-linking. After thorough washing with DI water, the achieved DCH-gel was soaked in 1 M Zn(OTf)_2_ for 12 h to obtain DCZ-gel. The Cel-gel was prepared without adding ECH while the other conditions kept unchanged. The mechanical tests of hydrogels are described in detail in the Supporting Information.

### Synthesis of PANI/Carbon Cloth (CC) Cathode

The PANI/CC cathode was synthesized by a typical in situ polymerization strategy [24]. First, 0.50 mL of aniline monomer was added to 20 mL of 1 M HCl solution with continuous stirring and then the CC pieces (25 mm × 25 mm) were dipped into the solution under an ice-water bath for 1 h. Next, 5.0 mL of 1 M HCl solution with 0.30 g of APS was added to the above solution dropwise at 0 °C. The polymerization continued for 1 h and the whole solution turned dark green. The PANI/CC cathode was washed with DI water and ethanol in turn, and then dried at 80 °C in a vacuum oven for 12 h. The PANI/CC pieces were then cut into small discs (12 mm in diameter). The mass loading of PANI on CC was about 1.0–1.5 mg cm^–2^.

### Characterization

Fourier transform infrared (FT-IR) spectra were recorded on a Nicolet 170-SX spectrometer (Thermo Nicolet, USA) in the wavenumber range from 4000 to 500 cm^–1^. Solid-state carbon nuclear magnetic resonance (^13^C NMR) spectra were conducted on a AVANCE NEO 400 (Bruker, USA). X-ray diffraction (XRD) measurements were performed on a Miniflex600 diffractometer (Rigaku, Japan). Using CuKα radiation (λ = 0.15418 nm) at 40 kV and 15 mA, the patterns were recorded in the 2θ region from 5° to 80° at a scanning speed of 5° min^–1^. Thermal gravimetric (TG) analysis was carried on TGA Q500 thermogravimetric analyzer (TA Instruments, USA) from 30 to 800 °C at a heating rate of 5 °C min^−1^ under an air atmosphere. X-ray photoelectron spectra (XPS) were recorded on a K-Alpha + spectrometer (Thermo Scientific, USA), and the PANI/CC pieces were sealed in Ar-filled aluminum–plastic bags before characterization. Field emission scanning electron microscopy (FE-SEM) observations and energy-dispersive X-ray spectroscopy (EDS) were performed on a MERLIN Compact microscopy (Zeiss, Germany). Both surface and cross-section of the dried specimens (frozen in liquid nitrogen, snapped immediately, and then freeze-dried) were coated with gold vapor. The light transmittance of DCZ-gel was measured using a UV–Vis spectrophotometer (Evolution 201, Thermo Scientific, USA) in the wavelength range from 300 to 800 nm. Topographic images of the surfaces of Zn anodes were observed on an atomic force microscopy (AFM, Asylum Research, UK) in dynamic contact mode. In situ observation of Zn dendrite growth was recorded using an Axio Vert.A1 microscope (Zeiss, Germany). All hydrogels were freeze-dried on a lyophilizer (Christ ALPHA, Germany) before characterization. The electrochemical measurements of the liquid and DCZ-gel electrolyte are described in detail in the Supporting Information.

## Results and Discussion

### Structure and Physicochemical Properties of DCZ-gel

**Fig. 1:**
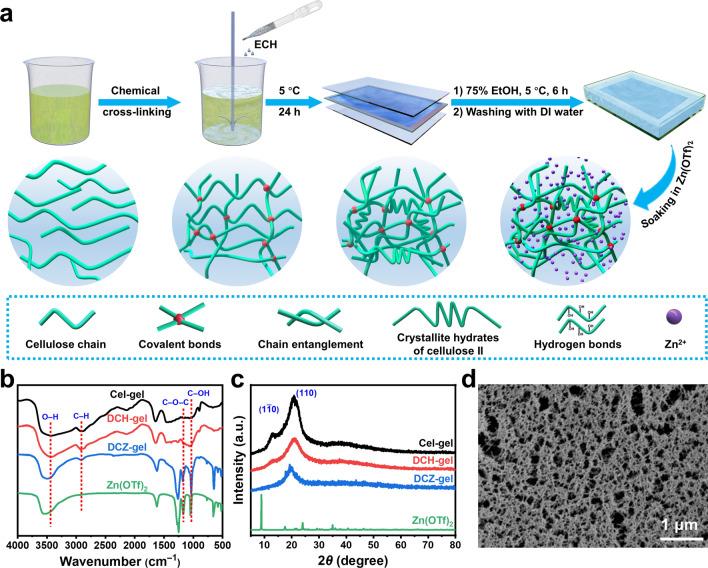
**a** Illustration of the preparation of DCZ-gel and the multiple interactions within the hydrogels. **b** FT-IR spectra and **c** XRD patterns of the cellulosic hydrogels and Zn(OTf)_2_. **d** Cross-sectional FE-SEM image of DCZ-gel

The DCZ-gel was fabricated via sequential chemical and physical cross-linking methods (Fig. 1a). In brief, cellulose was dissolved in an aqueous LiOH/urea solution to obtain transparent solution. Then, ECH, a commonly utilized base-catalyzed cross-linking agent for carbohydrate polymers, is employed to create a covalent network in here. Owing to the basicity of the aqueous LiOH/urea solvent system, the chlorine and epoxy groups on ECH could react with the –OH groups on the backbone of cellulose (the order reactivity is C6 > C2 > C3) via Williamson etherification and the alkali-catalyzed oxalkylation, respectively (Fig. S1) [25]. The precursor was subsequently immersed in a 75% aqueous ethanol solution to form physically cross-linked domains by hydrogen bonding and chain entanglements between cellulose chains, as well as crystallite hydrates of cellulose II. After thorough washing with DI water and soaking in Zn(OTf)_2_ aqueous solution, the DCZ-gel was obtained. The chemical structures of these hydrogels were characterized. In the FT-IR spectra (Fig. 1b), a series of characteristic peaks at 3443, 2914, 1169, and 1035 cm^−1^ attributed to the stretching vibration of O–H, C–H, C–O–C, and C–OH, respectively. The XPS spectra (Fig. S2b) showed the content of C–C/C–H increased while the content of C–O–C/C–O–H decreased after chemical cross-linking, proving the ECH can react with the –OH groups of cellulose to form covalent bonds. Besides, no Cl 2*p* peak was observed in the XPS spectra of DCH-gel, proving that the ECH and its chlorine has been completely removed (Fig. S2a). In the solid-state ^13^C NMR spectra of the Cel-gel and DCH-gel (Fig. S2c), the characteristic peaks of C4 and C6 showed a larger loss of resolution and shoulder of the broadening region, indicating reduced crystallinity of DCH-gel due to the disrupted intramolecular hydrogen bonding between the cellulose chains [26]. In addition, the XRD pattern of Cel-gel exhibited two strong peaks at 12.7° and 20.9° (Fig. 1c), which corresponded to the (1$\stackrel{\mathrm{-}}{1}$0) and (110) reflections of cellulose II crystallite, respectively. And the (110) reflection still appeared in DCH-gel and DCZ-gel, proving the existence of cellulose II crystallite hydrates [27]. Besides, the peaks of Zn(OTf)_2_ were absent in the DCZ-gel electrolyte, suggesting that Zn^2+^ and OTf^−^ ions are uniformly distributed within the hydrogel matrix [28].

The morphology of the hydrogels was observed by FE-SEM. In Fig. 1d, owing to the formation of appropriate chemical cross-linking, a hierarchically porous structure was realized in DCZ-gel. The pores in this structure could act as ion migration channels and thus facilitate the transport of Zn^2+^ ions, resulting in the enhancement of ionic conductivity [29]. In contrast, the Cel-gel showed a denser structure than those of DCH-gel and DCZ-gel due to the insufficient cross-linking within hydrogel network (Fig. S3a–c). The volume and corresponding concentration of Zn(OTf)_2_ aqueous solution in DCZ-gel was about 110 μL (for 16 mm disk in diameter) and 0.87 mol L^−1^, respectively, which was calculated by water content and residual weight measured by TG analysis, proving an excellent penetrability. Moreover, the corresponding energy dispersive spectroscopy (EDS) mapping images showed that the Zn^2+^ ions were uniformly distributed in the matrix of DCZ-gel (Fig. S3d, e).

**Fig. 2:**
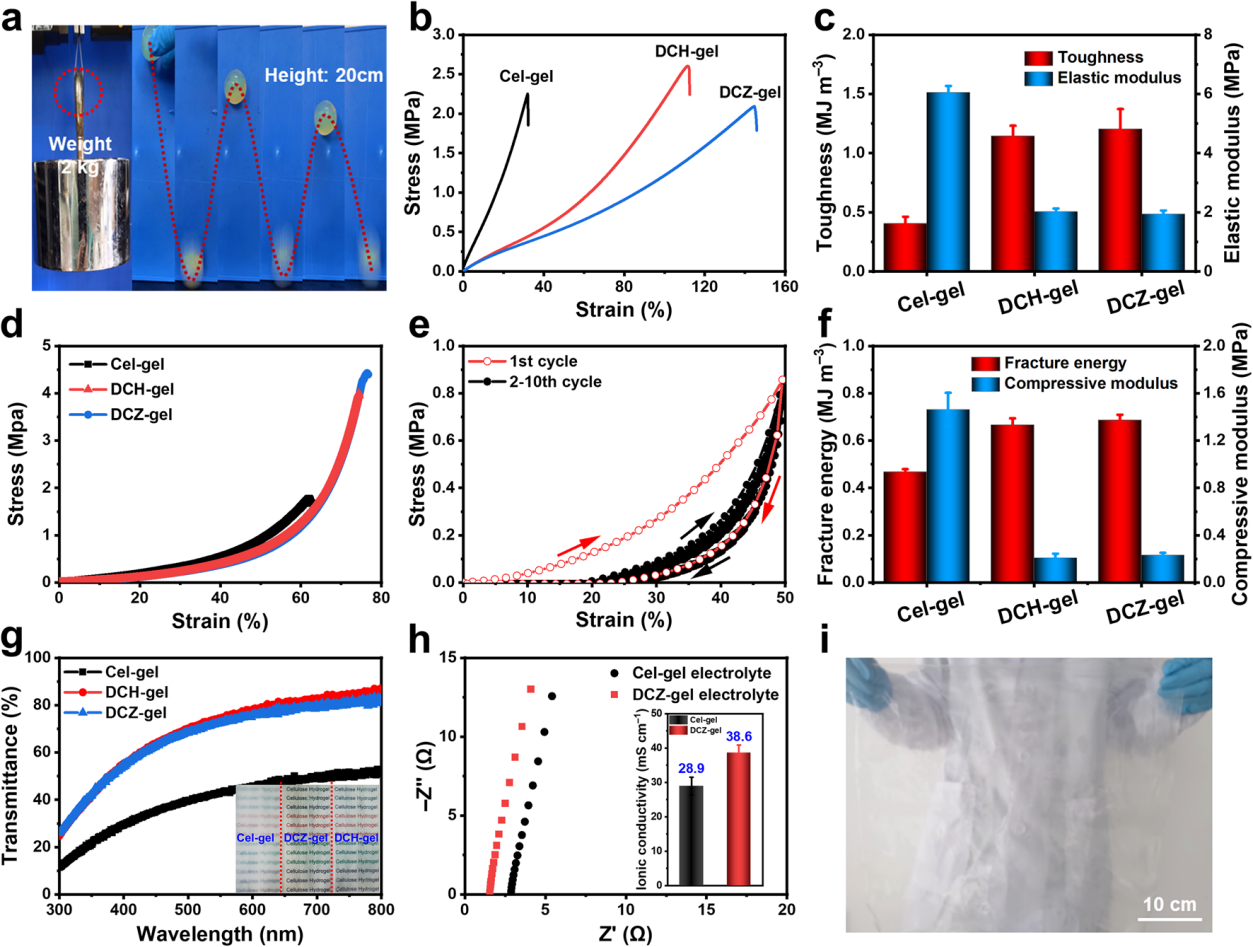
**a** Photographs of the rigidity and elasticity behaviors of DCZ-gels. **b** Tensile stress–strain curves of the cellulosic hydrogels, and **c** the corresponding toughness and elastic modulus. **d** Compressive stress–strain curves of the cellulosic hydrogels. **e** Successive loading–unloading curves under 50% compressive strain for 10 cycles. **f** The corresponding fracture energy and compressive modulus. **g** Light transmittance of the cellulosic hydrogels. **h** Ionic conductivities of Cel-gel and DCZ-gel from alternating current impedance curves. **i** Photograph of the large-area of DCZ-gel (50 cm × 50 cm)

The mechanical properties of hydrogels are key factors for the capacity to suppress Zn dendrites growth and the adaptability to sustain external forces and deformations [30]. Benefiting from the unique DC structure, the DCZ-gel exhibited high rigidity and elasticity. As displayed in Fig. 2a, the DCZ-gel could lift steel blocks with a weight of 2 kg and bounce back up to about 80% after falling free from a height of 20 cm. Then, the mechanical properties of the hydrogels were quantitatively investigated by tensile tests (Fig. 2b). The DCZ-gel (2.08 MPa, 145%) exhibited a similar tensile stress and higher tensile strain than DCH-gel (2.60 MPa, 111%) and Cel-gel (2.25 MPa, 32%), which might be due to the role of chemical cross-linking and Zn^2+^ ions coordination while maintaining internal physically cross-linked domains. Therefore, the toughness and elastic modulus of DCZ-gel reached 1.20 MJ m^−3^ and 1.93 MPa (Fig. 2c), respectively. Also, the relationship between the dosage of ECH and mechanical properties of DCZ-gel was studied (Fig. S4a, b). As the volume of the ECH increased, the toughness of the cellulosic hydrogels increased while their elastic modulus decreased. However, the toughness reached a maximum when the volume exceeded 3 mL, which was a typical feature of the DC network [31]. That is, the microstructure of the cellulose hydrogel was controlled by both the density of chemical and physical cross-linking, balancing them is crucial for improvement of the mechanical properties. A similar trend was observed for the compressive behavior of the cellulosic hydrogels in Fig. 2d. The DCZ-gel (4.42 MPa, 76%) exhibited higher compressive stress and strain than DCH-gel (4.00 MPa, 74%) and Cel-gel (1.77 MPa, 62%). Moreover, successive loading–unloading tests were performed at a maximum compressive strain of 50%, and the curves in different cycles almost overlapped with each other after the first cycle (Fig. 2e), suggesting that no prominent plastic deformation or strength degradation occurred. The corresponding fracture energy and compressive modulus also reached 0.68 MJ m^−3^ and 0.23 MPa (Fig. 2f), respectively, overcoming the intrinsic fragile properties of cellulose-based hydrogel. Therefore, the structural integrity of DCZ-gel could be retained during the assembly process of coin cells [32]. Likewise, increasing the ECH content reduced the compressive modulus of the cellulose hydrogel, and the fracture energy reached a maximum when the volume exceeded 2 mL (Fig. S4c, d). Notably, DCZ-gel exhibited other unique properties. As shown in Fig. 2g, due to the reduction of recrystallization of cellulose chains, the transmittance of DCZ-gel could reach 74.1% at 550 nm. Such a transparency in the visible light region could ensure the alignment of electrodes, which have a great impact on the electrochemical performance [33]. Attributing to the more ion transport channels, DCZ-gel had an ionic conductivity of 38.6 mS cm^−1^ calculated from the Nyquist plot in Fig. 2h, which was higher than that of Cel-gel (28.9 mS cm^−1^). Moreover, we evaluated the transference number of Zn^2+^ ions (${\text{t}}_{{\text{Zn}}^{2+}}$) of DCH-gel based on chronoamperometry (CA) and impedance curves (Fig. S5). A higher ${\text{t}}_{{\text{Zn}}^{2+}}$ of 0.73 was obtained, indicating its outstanding Zn^2+^ transport capability. Particularly, a large scale (50 cm × 50 cm) of DCZ-gel could be manufactured (Fig. 2i), highlighting its scalability and transparency.

### Electrochemical Performances of Zn Electrodes

**Fig. 3:**
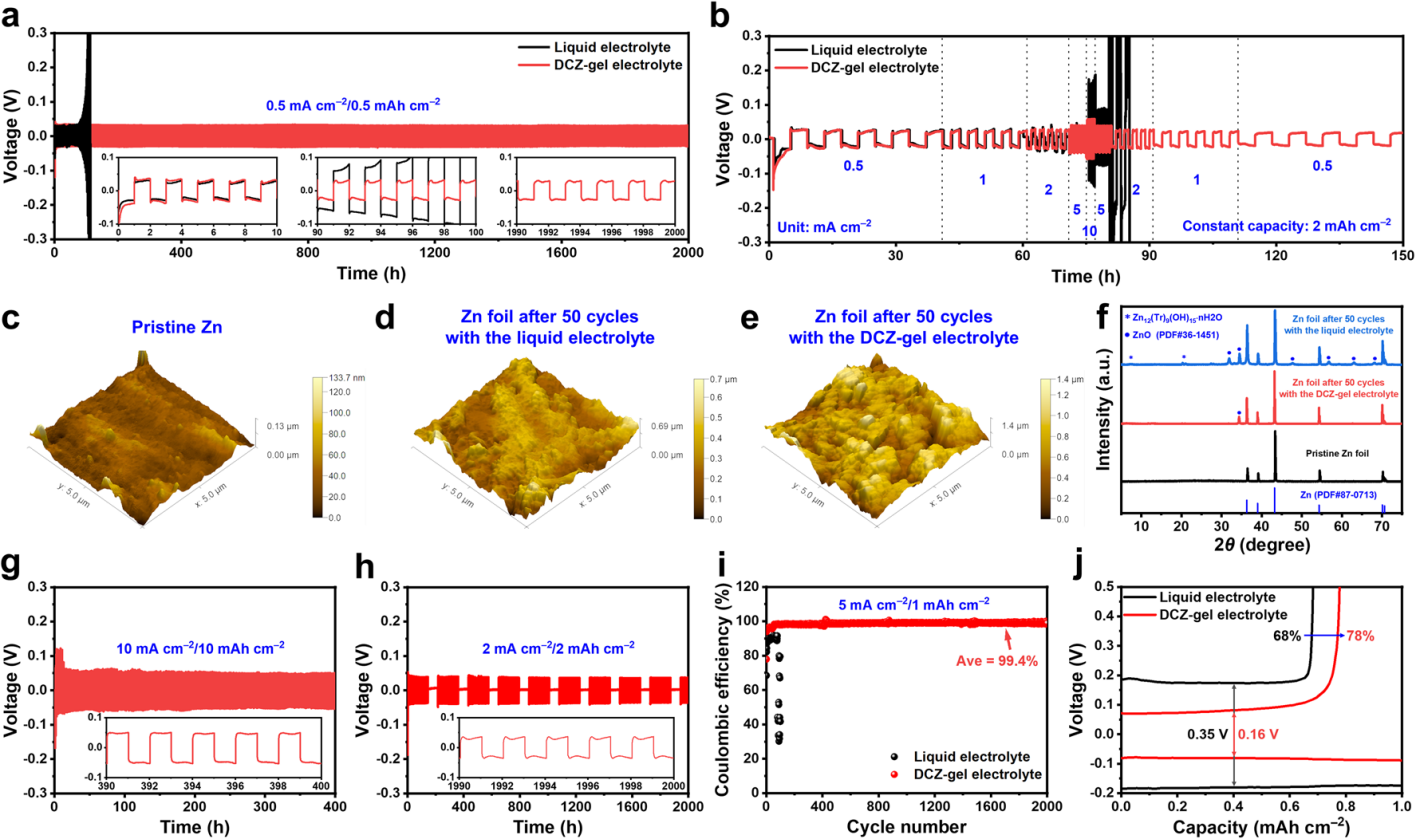
Voltage–time profile comparison of Zn||Zn cells using the liquid and DCZ-gel electrolytes at **a** current density and capacity of 0.5 mA cm^−2^/0.5 mAh cm^−2^, and **b** variated current densities with a capacity of 2 mAh cm^−2^. AFM images of Zn foils at **c** the pristine state and the deposited states after cycling (0.5 mA cm^−2^/0.5 mAh cm^−2^) for 50 cycles in Zn||Zn cells with the **d** liquid and **e** DCZ-gel electrolytes. **f** The corresponding XRD patterns of the Zn foils after cycling. Cycling performance of Zn||Zn cells under **g** large current density and capacity of 10 mA cm^−2^/10 mAh cm^−2^, and **h** alternating test between Zn plating − stripping cycling (72 cycles, 2 mA cm^−2^/2 mAh cm^−2^) and resting (72 h). **i** Coulombic efficiency profiles of Zn||Cu cells with the two electrolytes at 5 mA cm^−2^/1 mAh cm^−2^ and **j** the corresponding voltage–capacity profiles

Given the favorable properties mentioned above, DCZ-gel could be applicable as hydrogel electrolyte for ARZBs. DCZ-gel with 3 mL of ECH was cut into disks (with a diameter of 16 mm and a thickness of 0.71 mm, Fig. S6) to serve as the hydrogel electrolyte. Glass fiber (GF) separators (16 mm in diameter) with of 100 μL 1 M Zn(OTf)_2_ aqueous solution were used for comparison (i.e., liquid electrolyte). Firstly, we compared the Zn plating/stripping performance of Zn||Zn symmetric cells using the DCZ-gel electrolyte and liquid electrolyte at a current density of 0.5 mA cm^−2^ with an areal capacity of 0.5 mAh cm^−2^. The cell with the DCZ-gel electrolyte kept stable for over 2000 h, while the one with the liquid electrolyte could only cycle for less than 100 h with severe polarization (Fig. 3a). This might be because the Zn dendrites and side reactions damaged the interface between Zn electrode and electrolyte. Moreover, as shown in Fig. 3b, the Zn||Zn cell with the DCZ-gel electrolyte kept stable at increased current densities (from 0.5 to 10 mA cm^−2^) with a constant areal capacity of 2 mAh cm^−2^, but the cell with the liquid electrolyte showed voltage fluctuation from 2 mA cm^−2^. All these results indicated the excellent cycling lifespan and rate performance of Zn||Zn cell with the DCZ-gel electrolyte compared to that with the liquid electrolyte.

Subsequently, to reveal the Zn deposition, the morphology of Zn electrodes after 50 cycles at a fully deposited state was studied through AFM observation (Fig. 3c‒e). Clearly, the Zn surface with the liquid electrolyte was much rougher and bumpier than those at the pristine state and with the DCZ-gel electrolyte. The following FE-SEM characterizations (Fig. S7) confirmed that the Zn surface with the liquid electrolyte exhibited numberless mosslike dendrites, while that with the DCZ-gel electrolyte had a uniform plated Zn layer, indicating dendrite-free homogeneous Zn deposition. Moreover, the cross-sectional FE-SEM images demonstrated the difference in the new layer formed on the Zn electrode, which was loose and rugged for the liquid electrolyte (29.7 μm) but dense and flat for the DCZ-gel electrolyte (10.5 μm). Then, we performed ex situ XRD to analyze the composition of surface layer on Zn electrode (Fig. 3f). Lots of by-products such as Zn_12_(OTf)_9_(OH)_16_⋅nH_2_O and ZnO (PDF#36-1451) were formed on the Zn surface with the liquid electrolyte after cycling, but no obvious by-products were found on the Zn surface with the DCZ-gel electrolyte. These results demonstrated that DCZ-gel electrolyte could effectively inhibit the Zn dendrites growth and parasitic side reaction.

To further assess the Zn plating/stripping behavior enabled by the DCZ-gel electrolyte, we tested its tolerance to larger current density and areal capacity. Even in the case of 10 mA cm^−2^/10 mAh cm^−2^, the Zn||Zn cell could maintain a stable cycling for over 400 h with a low voltage hysteresis of ~ 50 mV (Fig. 3g), outperforming not only the cells with the liquid and Cel-gel electrolytes (Fig. S8a, b), but also most of previously reported polysaccharide-based ARZBs (Table S1). We also evaluated the shelf life and recovery ability of Zn||Zn cells by alternating cycling and resting measurements at 2 mA cm^−2^/2 mAh cm^−2^ (Fig. 3h). The cell with the DCZ-gel electrolyte could provide a sensational cycling stability for 2000 h, while the cell with the liquid and Cel-gel electrolytes became polarized after 300 h (Fig. S8c, d), demonstrating a high stability during the storage process. Moreover, we measured the Coulombic efficiency (CE) of Zn||Cu asymmetric cells to assess the Zn plating/stripping reversibility. As illustrated in Fig. 3i, the cell with the DCZ-gel electrolyte delivered a stabilized average CE of 99.4% within 2000 cycles at a current density of 5 mA cm^−2^, whereas the cell with the liquid electrolyte deteriorated after 76 cycles. The typical voltage profiles showed a smaller charge–discharge voltage gap of 0.16 V compared with the liquid electrolyte (Fig. 3j). The CEs measured at different plating conditions also confirmed this result (Fig. S9). Similar to Zn||Zn cells, we used FE-SEM to examine the morphology of Cu electrodes at a fully deposited state after cycling. The Cu surface with the liquid electrolyte exhibited rough dendrites, while that with the DCZ-gel electrolyte displayed a smooth and dense Zn layer after 50 cycles (Fig. S10).

**Fig. 4:**
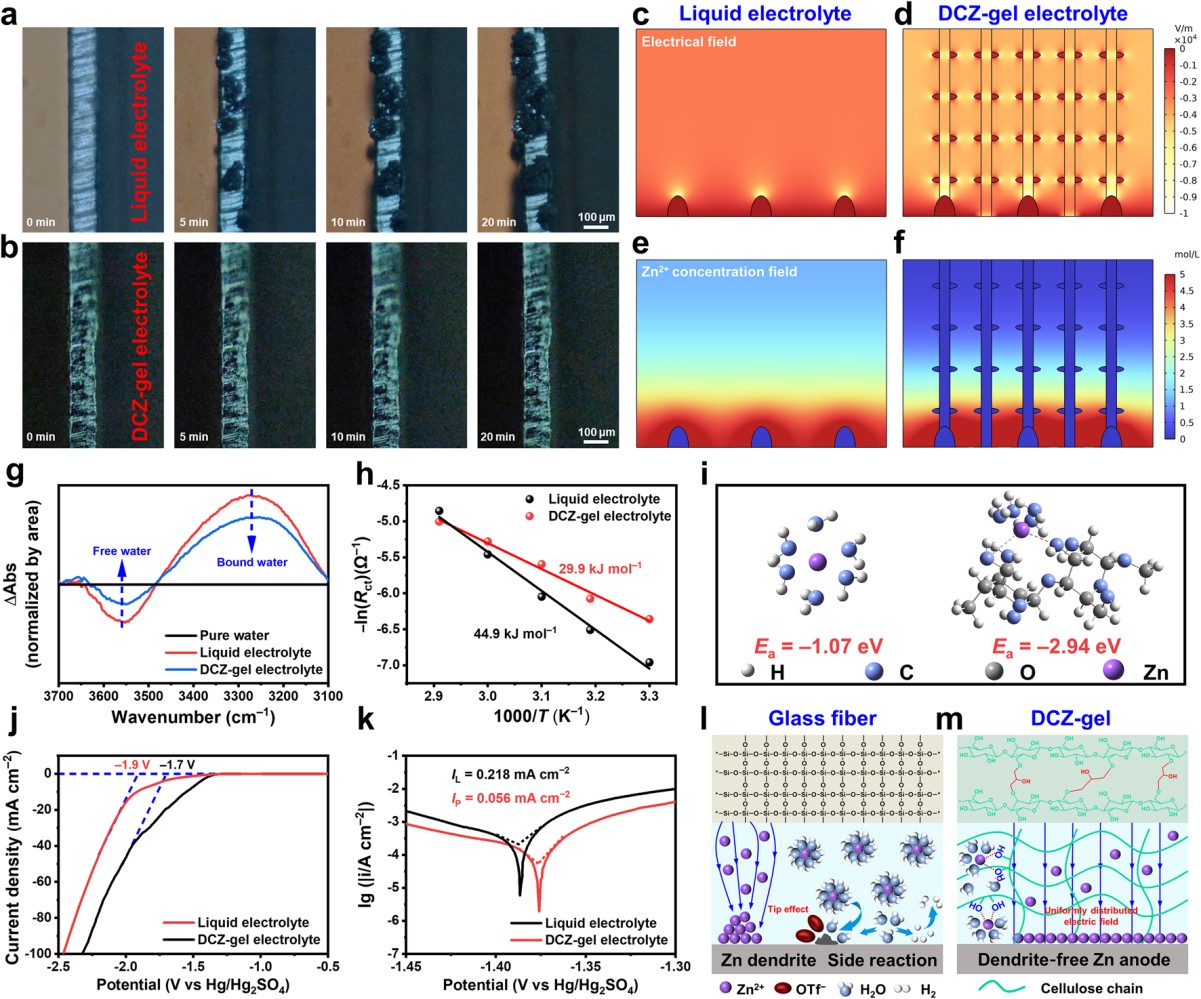
In situ optical microscopy observations of Zn electro-deposition process at 5 mA cm^–2^ for 20 min with **a** the liquid and** b** DCZ-gel electrolytes. COMSOL simulations of Zn electrode with **c, e** the liquid and **d, f** DCZ-gel electrolytes during plating **g** Differential ATR-FTIR spectra of the two electrolytes. **h** Arrhenius curves and the corresponding calculated desolvation activation energies. **i** Simplified models for DFT calculations of binding energies of Zn^2+^ ion in the two electrolytes. **j** LSV and **k** Tafel curves of the three-electrode cells with the liquid and DCZ-gel electrolytes at a scan rate of 1 mV s^−1^. Schematic diagrams of the proposed mechanisms on the Zn anode surfaces with **l** the liquid and **m** DCZ-gel electrolytes

Ulteriorly, astonished by the huge difference of performance between the two electrolytes, a series of methods were applied to intuitively and convincingly demonstrate the electro-deposition behavior of Zn^2+^ ions on Zn anode with different electrolytes. In situ growth process of Zn was recorded on an optical microscope, with a deposition duration of 20 min at a current density of 5 mA cm^–2^. Figure 4a shows that Zn dendrites emerged on the Zn surface with the liquid electrolyte within 5 min, and then rougher and thicker surface were observed after 20 min. As for the DCZ-gel electrolyte, the Zn surface remained smooth throughout the process (Fig. 4b), confirming the uniform Zn^2+^ ions flux for plating. Meanwhile, the nucleation and deposition process on Zn surface were also demonstrated by COMSOL simulations (Fig. S11), which could capture the interfacial electric field and Zn^2+^ ions flux variation [34]. As presented in Fig. 4c, the bare Zn electrode exhibited uneven electric field intensity at the initial nucleation, resulting in the “tip effect” that would induce the excessive deposition of Zn at the tips and thus facilitate the formation of sharp dendrites [35]. With the protection of the DCZ-gel electrolyte, the –OH groups along the hydrogel skeleton could homogenize the distribution of interfacial electric field and Zn^2+^ ions flux (Fig. 4d). The initial Zn nuclei uniformly distributed on the surface and caused the subsequent stable Zn plating. Subsequently, the Zn^2+^ ion concentration field on the electrode surface was calculated. The bare Zn electrode displayed a noticeable concentration gradient of Zn^2+^ ion between the electrolyte and electrode interface, as well as locally increased Zn^2+^ ion flux at the protuberances (Fig. 4e). In contrast, due to the abundant Zn^2+^ ions migration channel within the DCZ-gel electrolyte, the cells show a homogenous and strengthened Zn^2+^ ion flux near the electrode surface (Fig. 4f). Such Zn^2+^ ion flux could provide a fast and uniform supply of Zn^2+^ ion during Zn deposition [36]. Meanwhile, the electro-deposition behavior of Zn electrode was also proved by CA as presented in Fig. S12. When a constant voltage of 150 mV was applied, the current increased rapidly within 120 s for liquid electrolyte, indicating a prolonged and uncontrolled 2D planar diffusion [37]. In comparison, the 2D diffusion process ended within 50 s for DCZ-gel electrolyte and then a stable 3D diffusion occurred in the subsequent process.

The solvation structures of Zn^2+^ ions in liquid and DCZ-gel electrolytes were examined via the differential FT-IR spectra (Fig. 4g). More free water was observed in the DCZ-gel electrolyte than those in liquid electrolyte, verifying that –OH groups on the cellulose backbone captured a certain amount of desolvated Zn^2+^ ions and released the water molecules coordinated with Zn^2+^ ions [38]. The desolvation activation energies (*E*_a_) of hydrated Zn^2+^ ions were calculated from the Arrhenius equation to be 44.9 and 29.9 kJ mol^–1^ for liquid and DCZ-gel electrolytes (Figs. 4h, S13 and Table S3), respectively. This indicated that the DCZ-gel electrolyte could remove water sheath in [Zn(H_2_O)_6_]^2+^ and facilitate the desolvation process. It was further supported by DFT calculations (Fig. 4i and Fig. S14), which revealed that the binding energy of Zn^2+^ ions with –OH groups (− 2.94 eV) was higher than that with H_2_O (− 1.07 eV). Therefore, Zn^2+^ ions preferred to be attached by –OH groups rather than water and then form a stable bonding structure. Moreover, the corrosion behaviors of the Zn electrode with different electrolytes were also evaluated by using a three-electrode cell (Fig. 4j). The DCZ-gel electrolyte exhibited a lower corrosion current and higher corrosion voltage than the liquid electrolyte, verifying the low corrosion rate and tendency to the Zn electrode with the DCZ-gel electrolyte. Additionally, the linear sweep voltammetry (LSV) curves (Fig. 4k) verified a certain HER suppression effect of DCZ-gel electrolyte, as indicated by the 0.2 V lower HER potential compared to that for the liquid electrolyte. The COMSOL simulations confirmed the lower concentration and flux of H^+^ ions enabled by the DCZ-gel electrolyte (Fig. S15). Moreover, the electrochemical window was also wider than that of the liquid electrolyte (Fig. S16). These improvements owe a great deal to the stable bonding structure between the –OH groups of cellulose and Zn^2+^ ions.

Based on the above experimental results and theoretical analysis, we proposed a hypothesis for the mechanism of Zn plating/stripping with different electrolytes. For liquid electrolyte (Fig. 4l), Zn^2+^ ions will accumulate at regions of the electrode where the surface is rougher, creating uneven nucleation sites due to the higher concentrated electric field. Then, the Zn^2+^ ions will diffuse via the shortest path tends and form dendrites on the surface. Moreover, the H^+^ ions also accumulated to trigger H_2_ evolution, and then the consumption of H^+^ ions would facilitate water dissociation and increase the local concentration of OH^−^ anions, leading to Zn corrosion along with [Zn(H_2_O)_6_]^2+^. However, the DCZ-gel electrolyte with high strength and porosity can effectively homogenize the electric field and the Zn^2+^ ion flux (Fig. 4m). Furthermore, the –OH groups can bond with Zn^2+^ ions, which change the solvation structure and facilitates the desolvation process. Therefore, the DCZ-gel electrolyte can effectively suppress the formation of Zn dendrites and alleviate side reactions, thus enabling a stable Zn anode.

### Electrochemical Performances of Zn||PANI Cells

**Fig. 5:**
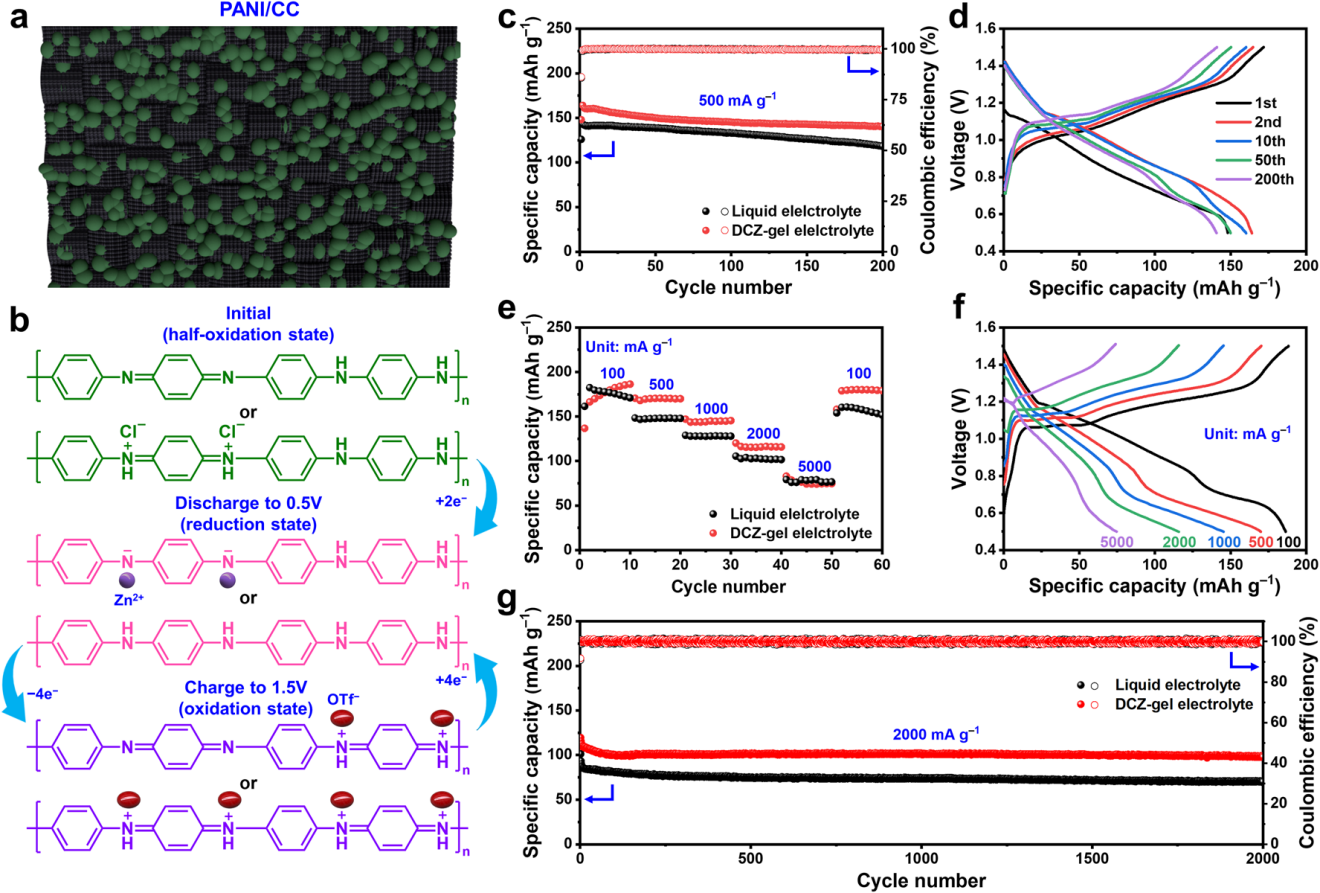
**a** Schematic diagram of the PANI/CC cathode, and **b** the corresponding charge/discharge mechanism. **c** Cycling performance at 500 mA g^–1^ and **d** the corresponding charge–discharge curves at different cycle numbers. **e** Rate performance and **f** the corresponding charge–discharge curves under different current rates from 100 to 5000 mA g^–1^. **g** Long-term cycling performance at 2000 mA g^–1^

The organic cathodes, such as PANI [39], phenazine [40], pyrene-4,5,9,10-tetraone [41], and sulfur heterocyclic quinones [42], emerge with tremendous potential for ARZBs benefiting from their easy structure designability and high theoretical capacity. The electrochemical redox reaction of them is based on the reversible charge state change of their electroactive functional group or moiety [43]. Among them, PANI containing long π-electron conjugated structure, could be as a promising candidate due to its high redox activity and fast kinetics conversion. On this basis, Zn||PANI cell was constructed to evaluate the application prospects of the DCZ-gel electrolyte. A PANI/CC cathode material was prepared by in situ polymerizing PANI on the surface of CC (Fig. 5a), and was presented the detailed structure characterization in Fig. S17. The intrinsic impact of the electrolytes on PANI redox process was investigated. Cyclic voltammetry (CV) was conducted for the Zn||PANI cells. The CV curves demonstrated two pairs of redox peaks located at 1.1/1.2 and 0.8/1.1 V vs. Zn^2+^/Zn (Fig. S18), respectively, corresponding to the successive two-step redox process in PANI [44]. Despite the similar CV curves for the liquid and DCZ-gel electrolytes, the integral area of the latter was larger than that of the former, indicating that the DCZ-gel electrolyte could improve the reaction kinetics of PANI to elevate the utilization of cathode active material. Theoretically, as shown in Fig. 5b, the pristine PANI is at a half-oxidation state, which will convert to a fully reduced and oxidized state when discharged to 0.5 V and charged to 1.5 V, respectively [45]. At the half-oxidation state, there are the oxidized N (e.g., − NH^+^ = , −NH^+^− , and −  = and reduced N (− NH –) along PANI chains, which are reduced to –N^−^– and –NH– groups during the discharge process, respectively. Meanwhile, the Cl^−^ will leave from the –NH^+^– groups, and the Zn^2+^ will interact with the –N^−^– groups. Then, in the following charge process, those –NH– groups are oxidized to –NH^+^–, which can interact with the OTf^−^ anions. The *ex-situ* XPS spectra at different charge/discharge steps (Fig. S19) proved the dual-ion storage mechanism in the whole redox process, where both Zn^2+^ and OTf^−^ participated in the charge storage process.

Based on the redox reaction mechanism of the PANI/CC cathode, the electrochemical performance of the DCZ-gel electrolyte in Zn||PANI cell was further studied (Fig. 5c). The cell with the DCZ-gel electrolyte posed a high reversible specific capacity of 160 mAh g^–1^ [59% of its theoretical capacity of 273 mAh g^–1^ (The doped anions are also taken into account)] at 500 mA g^−1^ and delivered a high-capacity retention of 88% after 200 cycles. Additionally, the average CE of the cell was as high as 99.74%, indicating the absence of side reactions. In comparison, the cell with the liquid electrolyte exhibited a lower reversible specific capacity of 141 mAh g^–1^ with a capacity retention of 83% after 200 cycles. The selected charge–discharge curves from the 1st to the 200th cycle exhibited two inconspicuous sloping platforms and showed much higher repeatability for the DCZ-gel electrolyte (Fig. 5d) than that for the liquid electrolyte (Fig. S20). Besides, the self-discharge behaviors of Zn||PANI cell were also investigated. After a rest of 48 h, the cell with the DCZ-gel electrolyte showed higher capacity retention compared to the liquid electrolyte (Fig. S21), indicating the excellent side reaction and corrosion resistance enabled by the DCZ-gel electrolyte. Moreover, the rate performance of Zn||PANI cell was evaluated under sequentially varied current rates (Figs. 5e, f and S22), and the specific capacities with the DCZ-gel electrolyte reached 186, 170, 145, 116, and 77 mAh g^−1^ at the last cycles of the steps under 100, 500, 1000, 2000, and 5000 mA g^−1^, respectively, which were significantly higher than those with the liquid electrolyte. When the current rate was reset to 100 mA g^−1^, the capacity could recover to 180 mAh g^−1^ (at the 60th cycle), confirming the superior redox reversibility. In addition, the long-term cycling performance under a high current rate of 2000 mA g^−1^ was investigated (Fig. 5g). The cell with the DCZ-gel electrolyte still delivered a reversible specific capacity of 108 mAh g^–1^ after 2000 cycles, corresponding to a high retention of 85%. These results suggested that the DCZ-gel electrolyte could not only alleviate the potential polarization of the Zn anode, but also suppress the structure deterioration of the PANI/CC cathode.

## Conclusion

In summary, we developed a sustainable DC cellulose hydrogel (DCZ-gel) electrolyte for a high-performance ARZBs. The DCZ-gel was fabricated using sequential chemical and physical cross-linking methods, leading to high mechanical strength and abundant ion migration channels. These characteristics enable the hydrogel outstanding dendrite suppression ability and enhanced ionic transport capacity. The DCZ-gel electrolyte achieved high ion conductivity of 38.6 mS cm^−1^, high Zn^2+^ ion transference number of 0.73, and homogenized electric field and Zn^2+^ ions flux for 3D diffusion to form dendrite-free Zn anode. Moreover, the –OH groups of cellulose could inhibit the formation of [Zn(H_2_O)_6_]^2+^, accelerate the desolvation of Zn^2+^ ions and suppresss the unfavorable side reactions including Zn corrosion and HER. As a result, the synergistic effects provided long cycling life (2000 h at 0.5 mA cm^−2^/0.5 mAh cm^−2^), high cumulative capability (400 h at 10 mA cm^−2^/10 mAh cm^−2^ with a low voltage hysteresis of 50 mV), and CE for Zn||Cu cell (averagely 99.4% within 2000 cycles at 5 mA cm^−2^/1 mAh cm^−2^), which outperformed the counterparts using liquid electrolyte and other polysaccharide-based hydrogel electrolytes so far reported. As a practical application of DCZ-gel electrolyte, the Zn||PANI cell exhibited a high reversible specific capacity of 160 mAh g^−1^ at 500 mA g^−1^, and a high-capacity retention of 85% after 2000 cycles at 2000 mA g^−1^. Therefore, this work opens a new avenue for developing high-performance cellulosic electrolytes toward green energy storage and conversion devices.

## Supplementary Information

Below is the link to the electronic supplementary material.

Supplementary file1 (PDF 2192 KB)
