# Supplementary material for: A Sustainable Dual Cross-Linked Cellulose Hydrogel Electrolyte for High-Performance Zinc-Metal Batteries
Source: Nanomicro Lett. 2024 Feb 2;16:106. doi: 10.1007/s40820-024-01329-0 (PMC10837397; doi:10.1007/s40820-024-01329-0)
Supplement: Supplementary file 1 — Supplementary file1 (PDF 2192 KB) [file 40820_2024_1329_MOESM1_ESM.pdf]

Supporting Information for

A Sustainable Dual Cross-Linked Cellulose Hydrogel Electrolyte for High-Performance Zinc-Metal Batteries

Haodong Zhang[1], Xiaotang Gan[2], Yuyang Yan[1], Jinping Zhou[1,*]

[1]Hubei Engineering Center of Natural Polymers-based Medical Materials, Key Laboratory of Biomedical Polymers of Ministry of Education, College of Chemistry and Molecular Sciences, Wuhan University, Wuhan 430072, P. R. China

[2]Hubei Key Laboratory of Electrochemical Power Sources, College of Chemistry and Molecular Sciences, Wuhan University, Wuhan, 430072, P. R. China

*Corresponding author. E-mail: zhoujp325@whu.edu.cn (J. Zhou)

S1 Experimental Section

S1.1 Mechanical Tests

All mechanical tests were performed at 30 °C and 50 % relative humidity using a universal tensile-compressive tester (Model 5576, INSTRON, USA) equipped with a 1000 N load cell. For tension, the hydrogel with square shape (length of 20 mm, width of 10 mm, and thickness of 0.70 mm) was measured at a rate of 10 mm min^{-1}. For compression, the hydrogel with cylindrical shape (height of 10 mm and diameter of 15 mm) was measured at a rate of 10 mm min^{-1}. The elastic/compressive modulus and toughness/fracture energy could be calculated from the stress−strain curves. The tensile stress (δ) is defined as the loading force (F) divided by the cross−sectional area (A_0) of the original sample ($\delta = F/A_0$). The tensile strain (ε) is defined as the deformed length divided by the original length [$\varepsilon = (L - L_0)/L_0$]. The toughness/fracture energy (W) was obtained by integrating the area underneath the stress−strain curve by the following equation:

$$W = \int_{\varepsilon_0}^{\varepsilon_f} \delta(\varepsilon)\, d\varepsilon \qquad \text{(S1)}$$

where ε_f and ε_0 are the initial strain and fracture strain, respectively. The elastic/compressive modulus was calculated according to the initial linear slope (0.1−1% strain) of the stress−strain curve. For recovery experiment, the hydrogel was initially compressed to a predetermined strain (50%) and then unloaded at the same speed (10 mm min^{-1}).

S1.2 Electrochemical Measurements

CR2032-type coin cells were assembled in air atmosphere for most electrochemical measurements. The 0.70-mm thick DCZ-gel film was cut into disks (diameter of 16 mm) to be used as the hydrogel electrolyte. When the liquid electrolyte (i.e., aqueous solution of 1 M $Zn(OTf)_2$) was used, a glass fiber membrane (GF/D, Whatman, diameter of 16 mm) was employed as the separator. The Zn||Zn, Zn||Cu, Zn||PANI coin cells were fabricated by using Zn foils (thickness of 200 μm and purity of 99.99%), Cu foils (thickness of 20 μm), and the PANI/CC cathode with the same diameter of 10 mm, as well as.

All the electrochemical measurements were operated at 25 °C unless otherwise specified. The charge−discharge measurements of the above cells were conducted on a battery testing system (CT2001A, LAND, China). The charge cutoff voltage was 0.5 V for the Zn||Cu cells, and the voltage window was 0.5−1.5 V for Zn||PANI. The cyclic voltammetry (CV), linear scan voltammetry (LSV),

chronoamperometry (CA), and electrochemical impedance spectroscopy (EIS) were carried out on an electrochemical workstation (1470E, Solartron analytical, USA). For EIS tests, the frequency range was from 100 kHz to 0.1 Hz and the voltage amplitude was 10 mV.

Tafel tests were conducted using a three-electrode system made up of Zn, Pt, and Hg/Hg_2SO_4 electrodes as working, counter, and reference electrodes, respectively. For testing the DCZ-gel electrolyte in the three-electrode system, the Zn electrode was wrapped with the hydrogel with a thickness of ~0.70 mm and then immersed in the liquid electrolyte (1 M $Zn(OTf)_2$). LSV measurements were conducted using the same device, and aqueous solutions of 1 M NaOTf and 1 M $Zn(OTf)_2$ were used for cathodic and anodic scan, respectively.

The ionic conductivities (σ) were obtained from EIS measurements and calculated by the following equation:

$$\sigma = \frac{l}{RA} \tag{S2}$$

where l, R, and A are the thickness, bulk resistance, and area of the hydrogel electrolyte, respectively. The transference numbers of Zn^{2+} ion ($t_{Zn^{2+}}$) were obtained from the EIS measurements of the Zn||Zn symmetrical cells before and after a polarization (CA test) under 20 mV for 1000 s, and calculated by the following equation:

$$t_{Zn^{2+}} = \frac{I_s\left(\Delta V - I_0 R_0\right)}{I_0\left(\Delta V - I_S R_S\right)} \tag{S3}$$

where I_0 and R_0 are the initial current and resistance before polarization, I_s and R_s are the steady-state current and resistance after polarization, and ΔV is the polarization voltage (20 mV for a Zn electrode). The desolvation process of Zn^{2+} is usually the rate-limiting step of Zn deposition, which can be expressed by the activation energy (E_a) in the Arrhenius equation:

$$\frac{1}{R_{ct}} = Ae^{-\left(\frac{E_a}{RT}\right)} \tag{S4}$$

where R_{ct} is the charge transfer resistance, A is the frequency factor, R is the gas constant, and T is the absolute temperature.

S1.3 *In situ* Observation of Zn Dendrite Growth

A homemade transparent Zn||Zn cell was designed for *in situ* observation of the Zn plating/stripping processes with the liquid and DCZ-gel electrolytes. Two Zn foils (10 mm × 50 mm, thickness of 100 μm) were fixed in a cuvette by insulating tape, and separated by the electrolyte with a thickness of 1 mm. The cells were tested using a chronopotentiometry (CP) method on an electrochemical workstation (CHI760E, Shanghai Chenhua, China) at a current density of 5 mA cm^{-2}, and simultaneously observed under an optical microscope.

S1.4 DFT Calculations

All DFT calculations were performed using Gaussian 16 software [S1]. The B3LYP/6-31g* level was chosen to compute the geometrical optimizations, electron configurations, and Gibbs free energies of all species [S1]. Vibrational frequency calculations were carried out at the same level in order to verify the optimized structures at the local minimum. To calculate the weak interaction between two molecules, the basis set superposition error (BSSE) was used to eliminate the basic

function of the two molecules overlapping in the complex system [S2]. In addition, the solvation effect was considered by employing SMD methods [S3], and water was used as the solvent. The binding energy (E_b) of Zn^{2+} ion with a specified molecule (M) was calculated by the following equation:

$$E_b = E(Zn^{2+}\text{-M}) - E(Zn^{2+}) - E(\text{M}) + E(\text{BSSE}) \tag{S5}$$

where M is $6H_2O$ or the simplified model molecule of cellulose chains, and $E(Zn^{2+}\text{-M})$, $E(Zn^{2+})$, E(B), and E(BSSE) are the Gibbs free energies of Zn^{2+}-M complex, Zn^{2+}, M, and BSSE, respectively.

S1.5 Finite Element Simulation

Finite element simulation was conducted to analyze the electric field and ionic concentration distribution in the liquid and DCZ-gel electrolyte. The models were simplified to a unit with a height of 18 μm and a width of 24 μm. The protrusions on the Zn surface were represented by semi ellipses with a long axis of 2 μm and a short axis of 1 μm. According to the structural characteristics of DCZ-gel electrolyte, its model with channels were constructed. The simulations were performed by COMSOL Multiphysics based on solving the Nernst-Planck equation. The ion migration and diffusion behavior driven by the established electric fields were considered during the process. The boundary conditions of cathode and anode were set as experimentally measured voltage hysteresis and a constant of 0 V, respectively. For the simulation of hydrogen evolution, the exchange current density parameter was introduced to solve the Butler-Volmer equation. The exchange current density of hydrogen evolution was obtained by experimental measurement and calculation.

Supplementary Figures

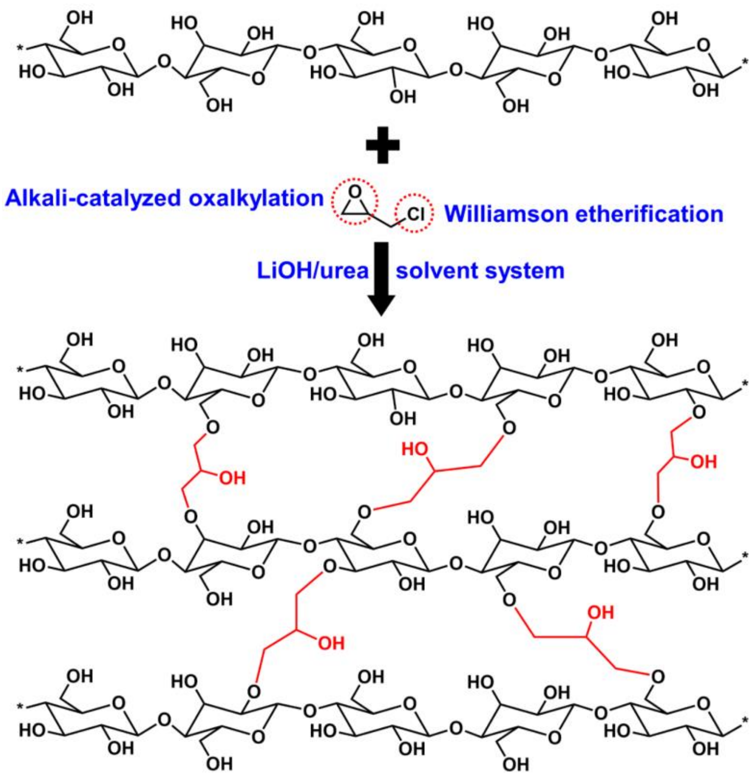

Fig. S1 The chemical structure of covalent network within DCZ-gel

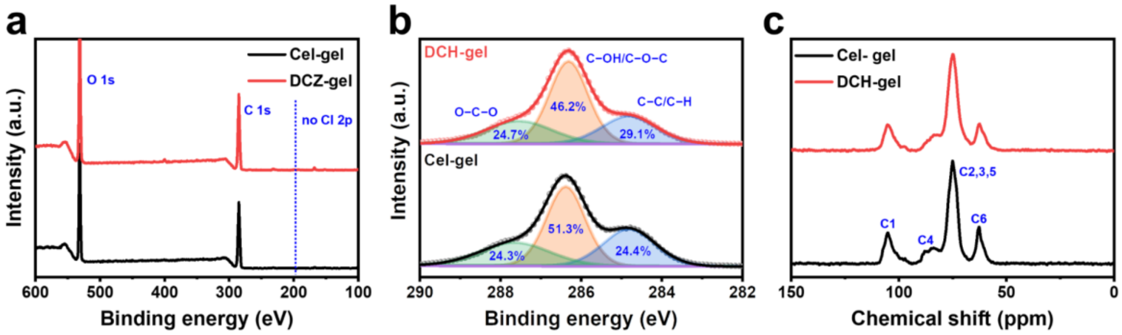

Fig. S2 a The full XPS spectra, **b** corresponding peak fitting of C1s, and **c** solid-state ^{13}C NMR spectra of the Cel-gel and DCH-gel

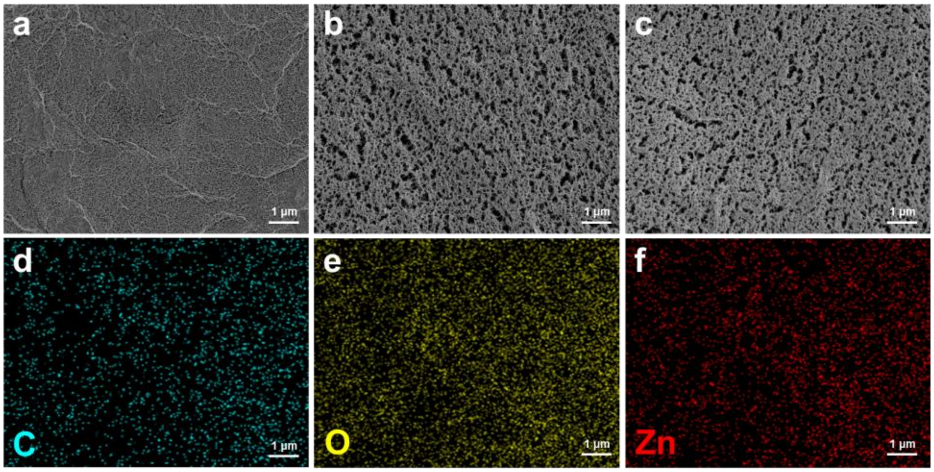

Fig. S3 Cross-sectional FE-SEM images of **a** Cel-gel, **b** DCH-gel, and **c** DCZ-gel. **d-f** EDS mapping images of DCZ-gel

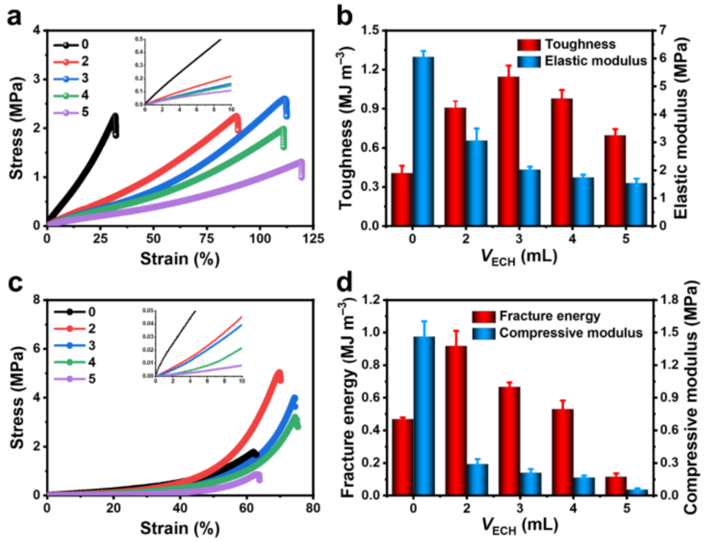

Fig. S4 a Tensile stress−strain curves (the inset is a local amplification Figure of the initial part), and **b** the corresponding toughness and elastic modulus; **c** compressive stress−strain curves (the inset is a local amplification Figure of the initial part), and **d** the corresponding fracture energy and compressive modulus of DCH-gel containing different amount of ECH

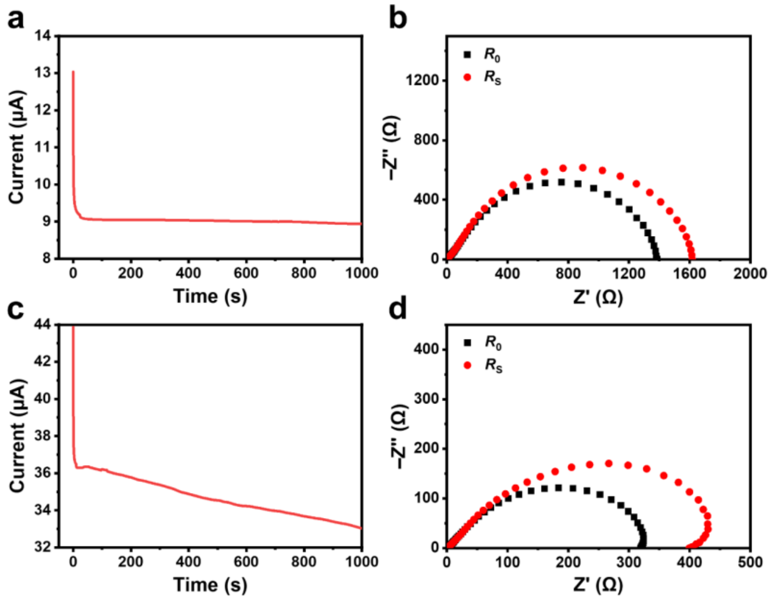

Fig. S5 CA curves (under 20 mV) and Nyquist plots at the initial and steady states of Zn||Zn cells with **a, b** the liquid and **c, d** DCZ-gel electrolytes for the calculations of transference numbers of Zn^{2+} ion

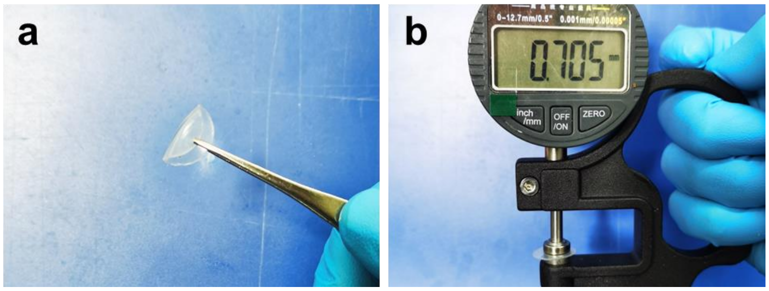

Fig. S6 a Photographs and **b** thickness measurement of the DCZ-gel electrolyte

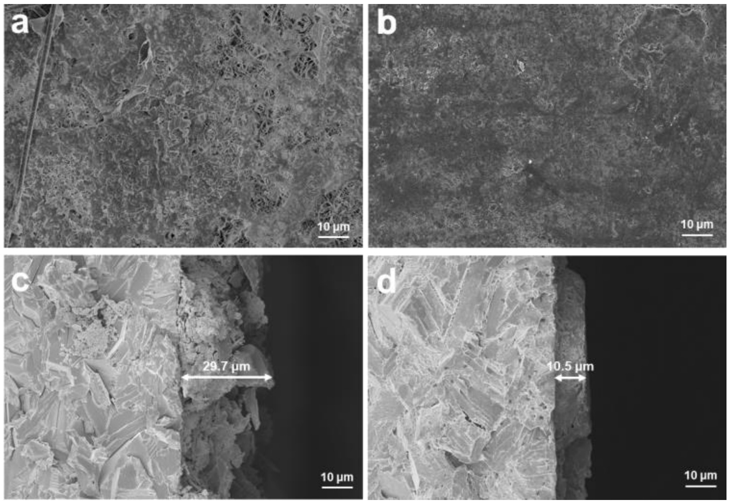

Fig. S7 a, b Surface and **c, d** cross-sectional FE-SEM images of Zn foils at deposited state after cycling (0.5 mA cm^{-2}/0.5 mAh cm^{-2}) after 50 cycles in the Zn||Zn symmetrical cells with **a, c** the liquid and **b, d** DCZ-gel electrolytes

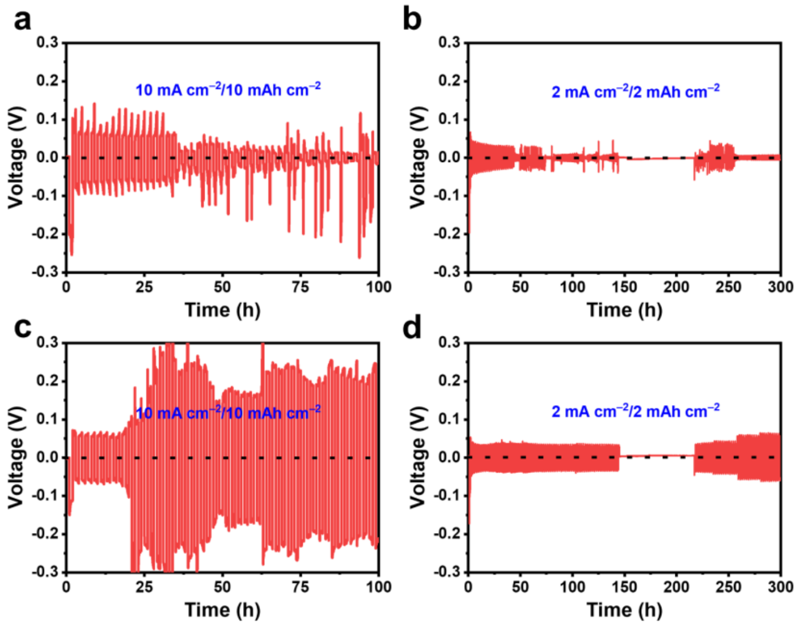

Fig. S8 Cycling performance of Zn||Zn cells under **a, c** large current density and capacity of 10 mA cm^{-2}/10 mAh cm^{-2} and **b, d** an alternating test between Zn plating−stripping cycling (72 cycles, 2 mA cm^{-2}/2 mAh cm^{-2}) and resting (72 h) with **a, b** the liquid and **c, d** Cel-gel electrolytes

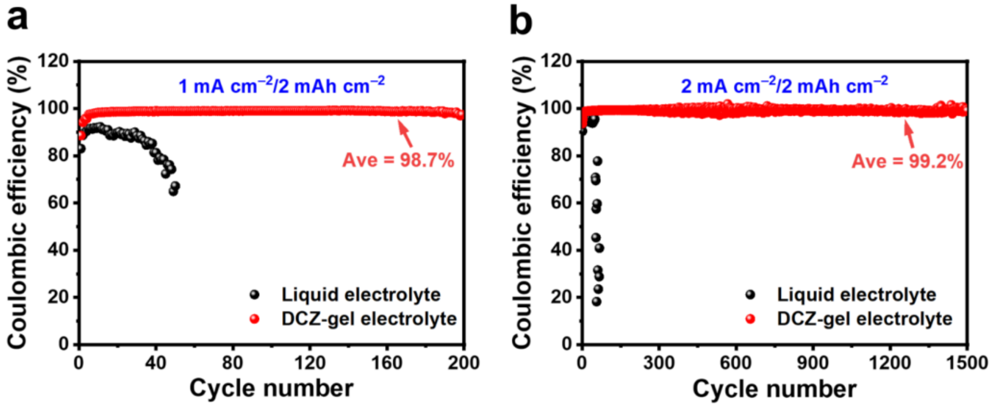

Fig. S9 Coulombic efficiency profiles of Zn||Cu cells with the liquid and DCZ-gel electrolytes at **a** 1 mA cm^{-2}/2 mAh cm^{-2} and **b** 2 mA cm^{-2}/2 mAh cm^{-2}

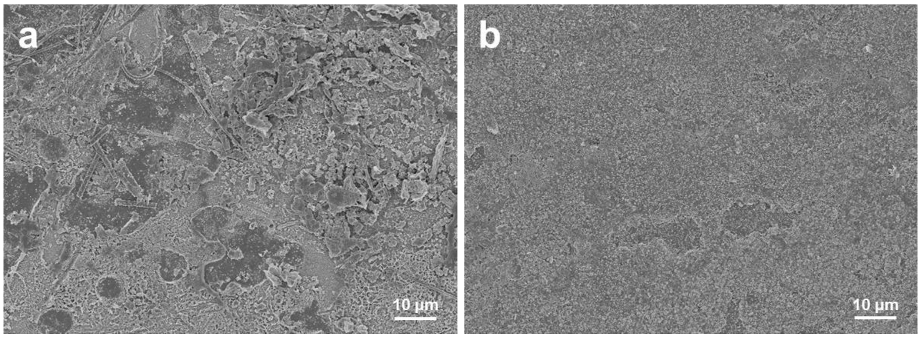

Fig. S10 Surface FE-SEM images of Cu foils at deposited state after cycling (0.5 mA cm^{-2}/0.5 mAh cm^{-2}) after 50 cycles in the Zn||Cu symmetrical cells with **a** the liquid and **b** DCZ-gel electrolytes

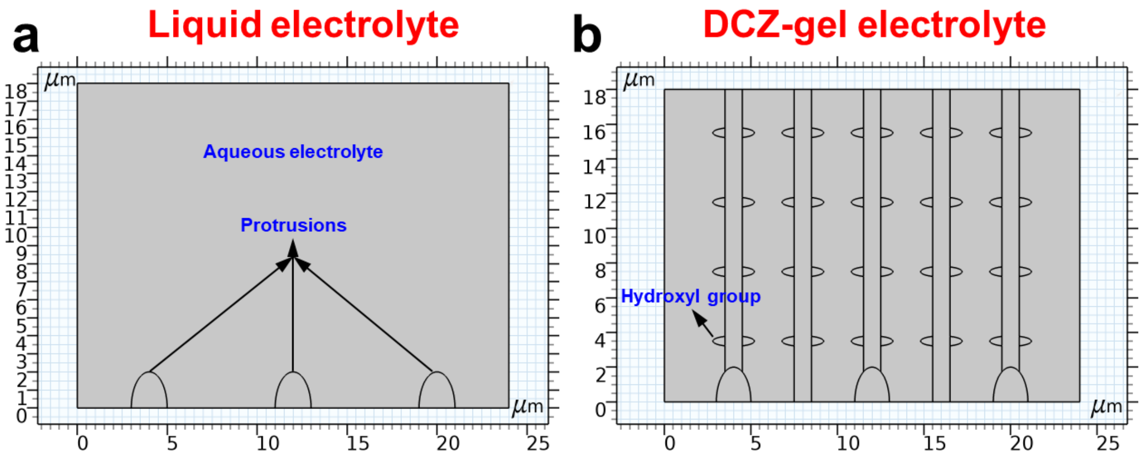

Fig. S11 Geometric models of the Zn electrodes in different electrolytes for simulations: **a** the liquid and **b** DCZ-gel electrolytes

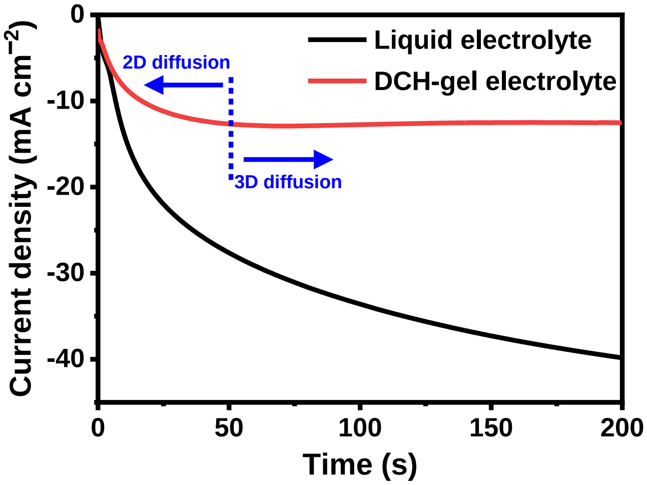

Fig. S12 CA curves of the Zn||Zn cells with the two electrolytes under a constant voltage of 150 mV

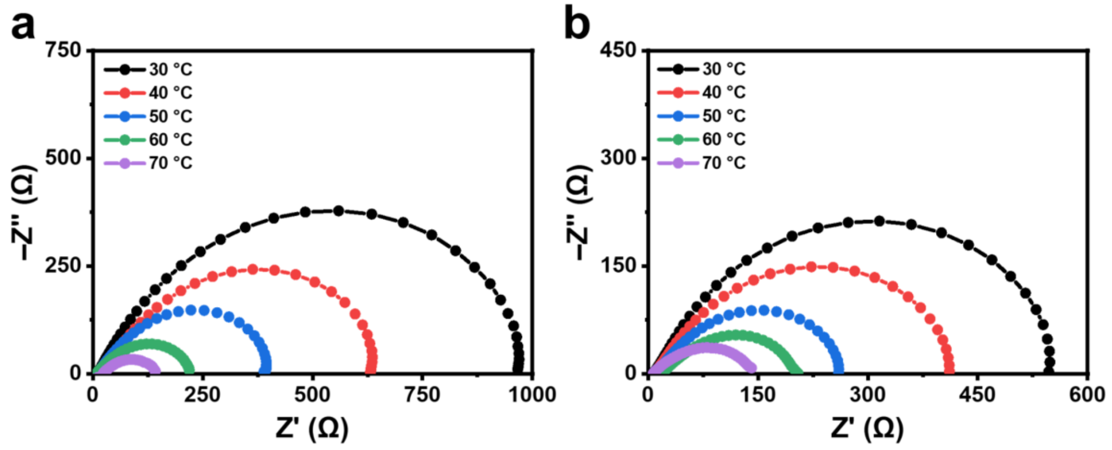

Fig. S13 Nyquist plots of the pristine Zn||Zn cells with **a** the liquid and **b** DCZ-gel electrolytes at different temperatures for the calculations of desolvation activation energies

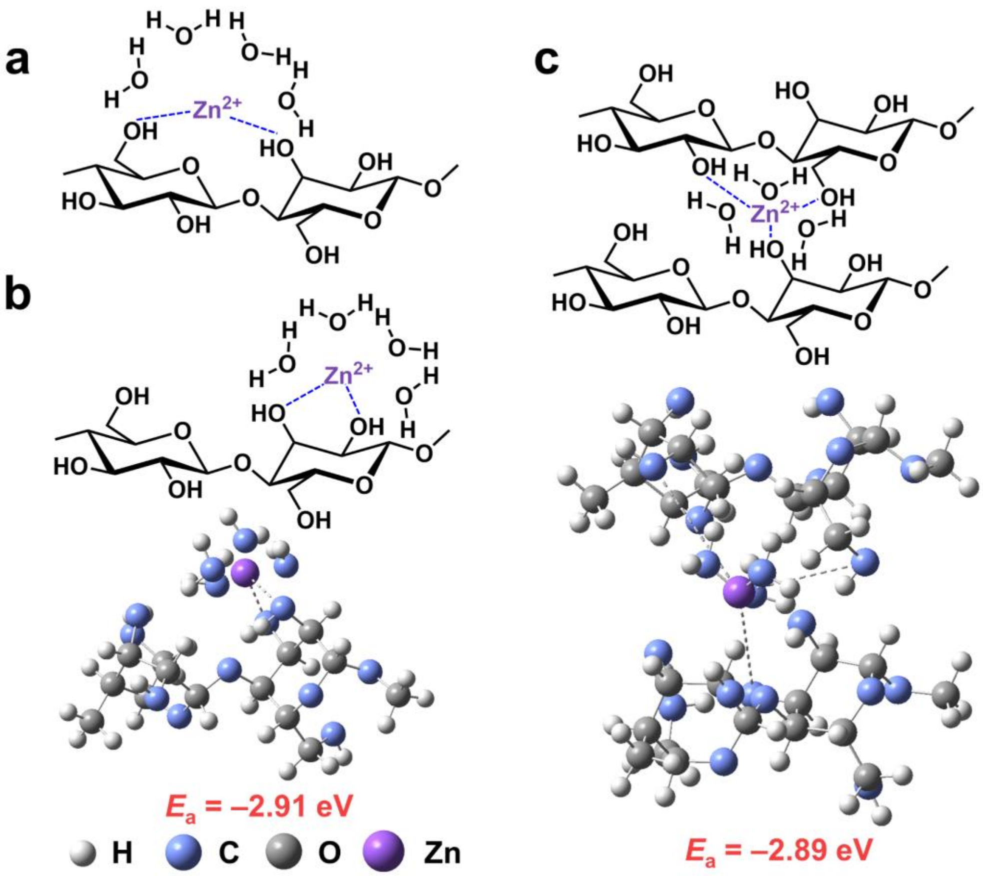

Fig. S14 The binding energy of cellulose C_2, C_3, and C_6 hydroxyl groups combined with Zn^{2+} ions

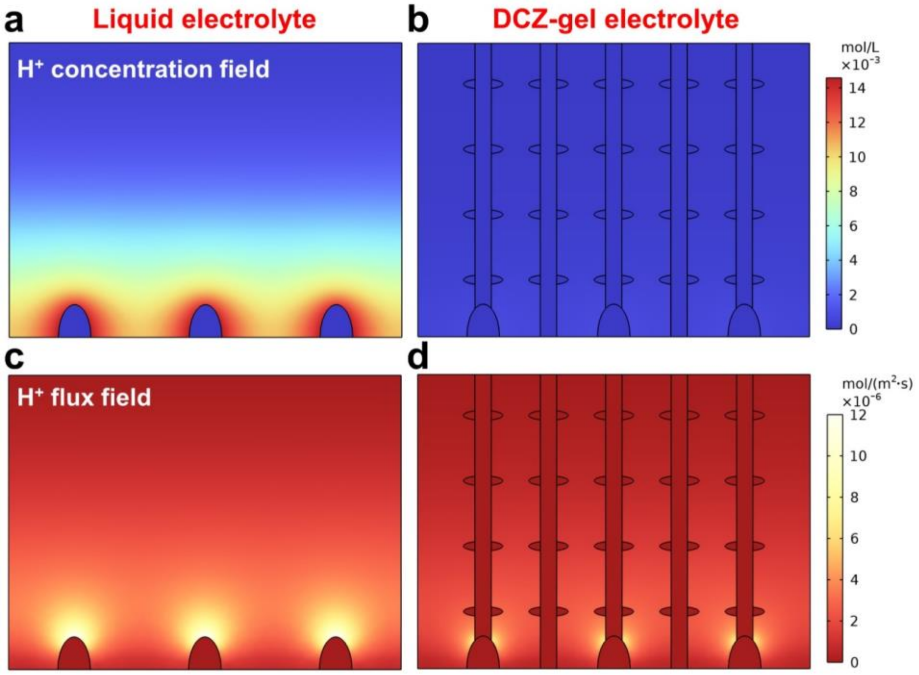

Fig. S15 Simulation of **a, b** H^+ concentration distribution and **c, d** H^+ flux after cycling in **a, c** liquid and **b, d** DCZ-gel electrolytes

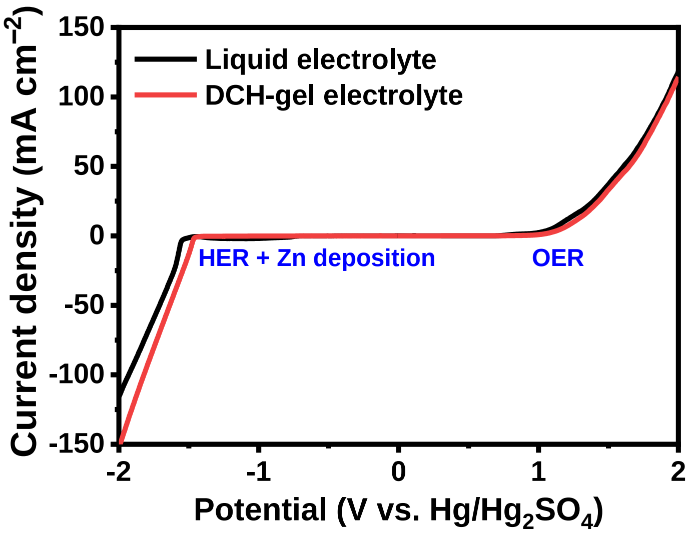

Fig. S16 LSV curves of the three-electrode cells with the liquid and DCZ-gel electrolytes at a scan rate of 1 mV s^{-1}

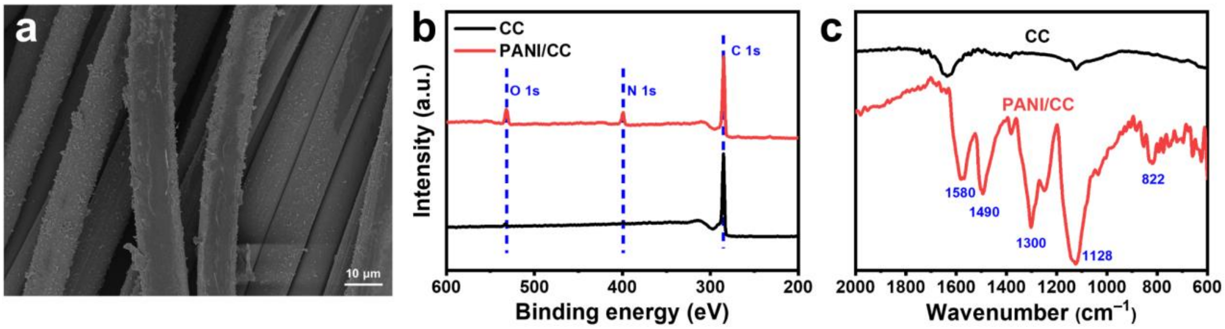

Fig. S17 a FE-SEM image, **b** XPS, and **c** FT-IR spectra of the PANI/CC cathode

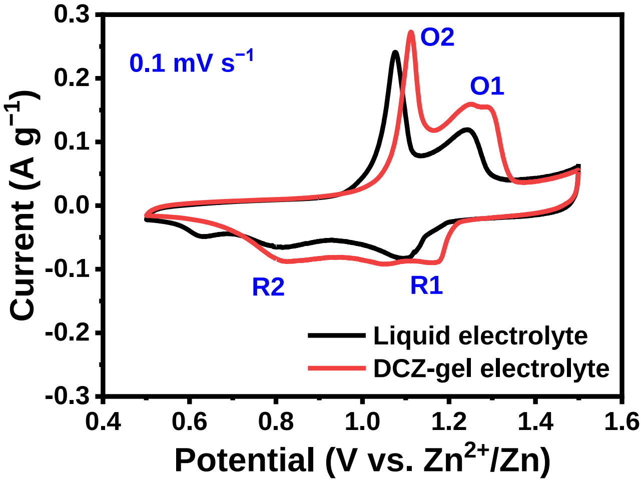

Fig. S18 Typical CV curves of Zn||PANI cell with the liquid and DCZ-gel electrolytes at a scan rate of 0.1 mV s^{-1}

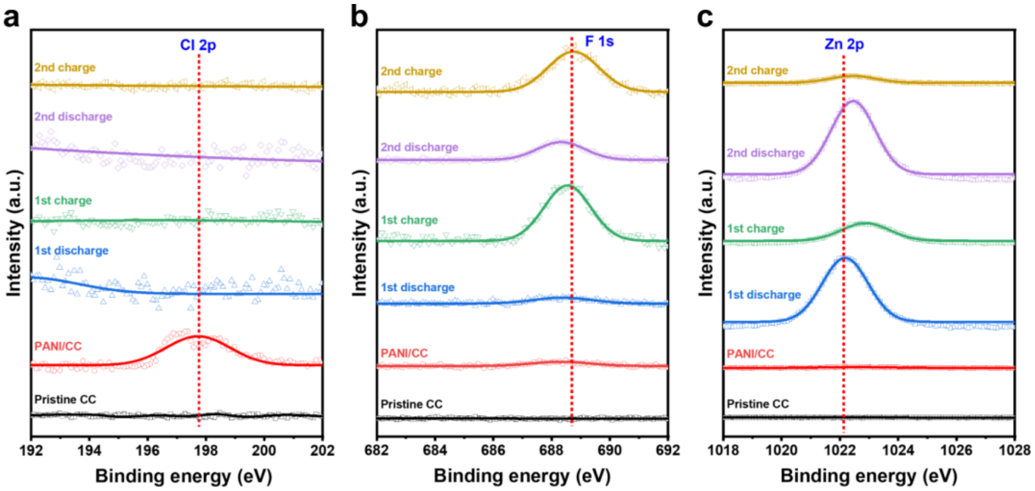

Fig. S19 XPS spectra of **a** Cl 2p, **b** F 1s, and **c** Zn 2p of PANI/CC cathodes at different states

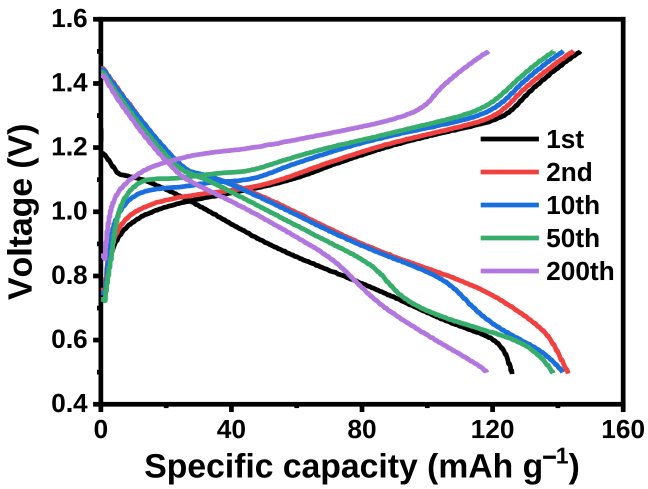

Fig. S20 Charge–discharge curves at different cycles under 500 mA g^{-1} of Zn||PANI cells with the liquid electrolyte

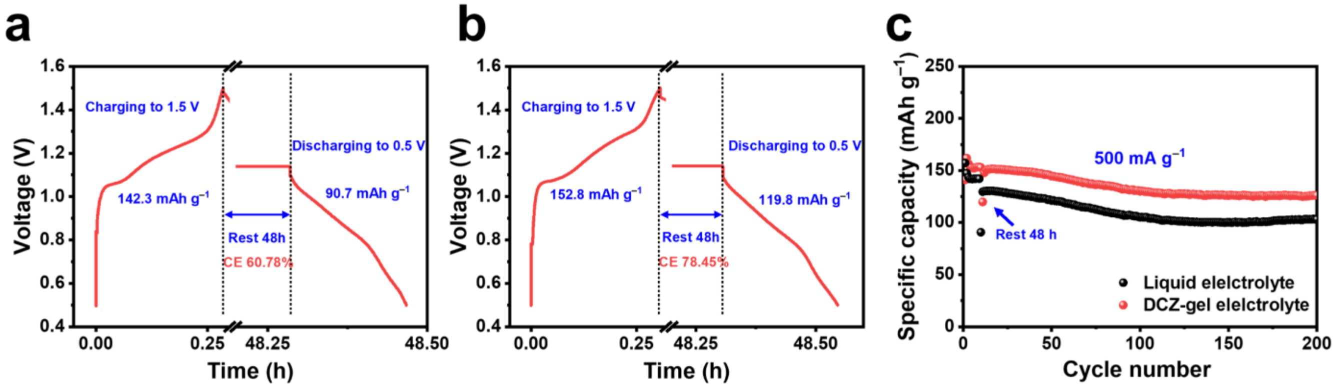

Fig. S21 Self-discharge test of Zn||PANI cells using **a** the liquid and **b** DCZ-gel electrolytes by resting for 48h at fully charged state after 10 cycles at 500 mA g^{-1}. **c** Cycling performance after resting for 48 h

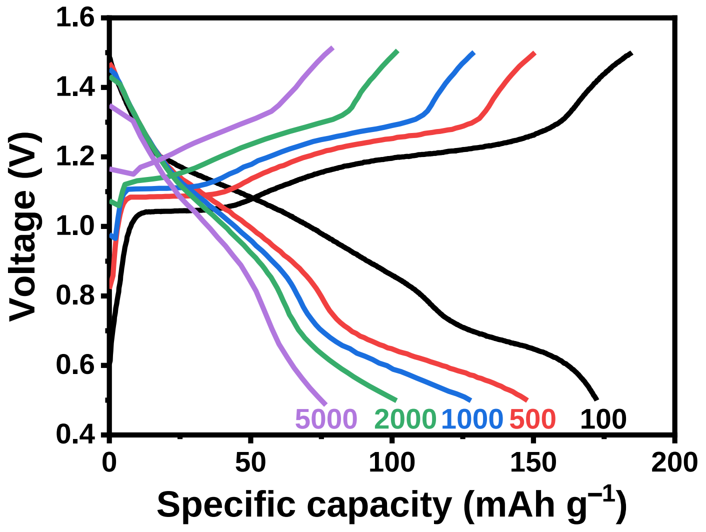

Fig. S22 Charge–discharge curves at different current rates of Zn||PANI cells with the liquid electrolyte

Supplementary Tables

Table S1 The electrochemical performances comparison between the DCZ-gel electrolyte and other reported polysaccharide-based hydrogel electrolytes for ARZBs

Refs.	Hydrogel electrolyte	Zn‖Zn cell		Zn‖Cu cell		Full cell		
		Current density/capacity (mA cm^{-2}/mAh cm^{-2})	Cycle life (h)	Current density (mA cm^{-2})	Average CE (%)/ Number of cycles	Cathode material	Reversible capacity (mAh g^{-1})/ Current density (A g^{-1})	Capacity retention (%)/ Number of cycles
**This work**	**DCZ-gel**	**0.5/0.5**<br>**10/10**	**2000**<br>**400**	**2**	**99.4/2000**	**PANI/CC**	**160/0.5**	**85/2000**
[S4]	Cellulose	10/5	350	10	99.5/500	MnO_2	200/2	95/500
[S5]		1/1	2400	——	——	MnO_2/CNT	100/6	99/500
[S6]		2/2	800	——	——	MnO_2/rGO	183/3	99/2000
[S7]		2/1	800	2	—/200	PANI/CC	80/1	75/1000
[S8]	Chitosan	10/10	1800	10	99.7/500	Poly(benzoquinonyl sulfide)	200/0.4	71/400
[S9]		5/1	450	1	99.6/400	MnO_2	180/1	98/1000
[S5]	Alginate	1/1	500	——	——	Active carbon	64/5	85/6000
[S10]		1/1	400	1	—/300	V_2O_5/CNT	250/2	85/200
[S11]		0.5/0.5	1600	1	1000	MnO_2	214/0.6	—/500
[S12]		1.1/1.1	300	1	100/200	$NH_4V_4O_{10}$	226/2	95/900
[S13]	Agarose	1/1	4000	——	——	Active carbon	252/0.3	90/400
[S14]	Guar gum	0.2/0.2	290	——	——	MnO_2/rGO	143/6	85/2000
[S15]	xanthan gum	——	——	——	——	MnO_2/CNT	282/0.3	90/330

Table S2 Parameters for the calculations of $t_{Zn^{2+}}$ of Zn||Zn cells with the liquid and DCZ-gel electrolytes obtained from Fig. S5

Electrolyte	I_0 (μA)	I_s (μA)	R_0 (Ω)	R_S (Ω)
Liquid	13	9	1324	1546
DCZ-gel	44	33	266	346

Table S3 R_{ct} values of Zn||Zn cells with the liquid and DCZ-gel electrolytes at different temperatures obtained from Fig. S13

Temperature (℃)	Liquid electrolyte (Ω)	DCZ-gel electrolyte (Ω)
30	1052	577
40	671	434
50	423	269
60	235	197
70	128	149

Supplementary References

[S1] G. RA. 1, mj frisch, gw trucks, hb schlegel, ge scuseria, ma robb, jr cheeseman, g. Scalmani, v. Barone, b. Mennucci, ga petersson et al., gaussian. Inc, Wallingford CT. **121**, 150-166 (2009).

[S2] S. F. Boys, F. Bernardi. The calculation of small molecular interactions by the differences of separate total energies. Some procedures with reduced errors. Mol Phys. **19**(4), 553-566 (1970). https://doi.org/10.1080/00268977000101561

[S3] A. V. Marenich, C. J. Cramer, D. G. Truhlar. Universal solvation model based on solute electron density and on a continuum model of the solvent defined by the bulk dielectric constant and atomic surface tensions. The Journal of Physical Chemistry B. **113**(18), 6378-6396 (2009). https://doi.org/10.1021/jp810292n

[S4] L. Xu, T. Meng, X. Zheng, T. Li, A. H. Brozena, Y. Mao, Q. Zhang, B. C. Clifford, J. Rao, L. Hu. Nanocellulose-carboxymethylcellulose electrolyte for stable, high-rate zinc-ion batteries. Adv Funct Mater. **33**(27), 2302098 (2023). https://doi.org/https://doi.org/10.1002/adfm.202302098

[S5] F. Cao, B. Wu, T. Li, S. Sun, Y. Jiao, P. Wu. Mechanoadaptive morphing gel electrolyte enables flexible and fast-charging zn-ion batteries with outstanding dendrite suppression performance. Nano Res. **15**(3), 2030-2039 (2022). https://doi.org/10.1007/s12274-021-3770-8

[S6] M. Chen, J. Chen, W. Zhou, X. Han, Y. Yao, C.-P. Wong. Realizing an all-round hydrogel electrolyte toward environmentally adaptive dendrite-free aqueous Zn–MnO_2 batteries. Adv. Mater. **33**(9), 2007559 (2021). https://doi.org/https://doi.org/10.1002/adma.202007559

[S7] Y. Quan, W. Zhou, T. Wu, M. Chen, X. Han, Q. Tian, J. Xu, J. Chen. Sorbitol-modified cellulose hydrogel electrolyte derived from wheat straws towards high-performance environmentally adaptive flexible zinc-ion batteries. Chem. Eng. J. **446**, 137056 (2022). https://doi.org/https://doi.org/10.1016/j.cej.2022.137056

[S8] M. Wu, Y. Zhang, L. Xu, C. Yang, M. Hong, M. Cui, B. C. Clifford, S. He, S. Jing, Y. Yao, L. Hu. A sustainable chitosan-zinc electrolyte for high-rate zinc-metal batteries. Matter **5**(10), 3402-3416 (2022). https://doi.org/https://doi.org/10.1016/j.matt.2022.07.015

[S9] X. Yang, W. Wu, Y. Liu, Z. Lin, X. Sun. Chitosan modified filter paper separators with specific ion adsorption to inhibit side reactions and induce uniform zn deposition for aqueous zn batteries. Chem. Eng. J. **450**(137902 (2022). https://doi.org/https://doi.org/10.1016/j.cej.2022.137902

[S10] W. Fan, Z. Sun, Y. Yuan, X. Yuan, C. You, Q. Huang, J. Ye, L. Fu, V. Kondratiev, Y. Wu. High cycle stability of zn anodes boosted by an artificial electronic–ionic mixed conductor coating layer. J. Mater. Chem. A **10**(14), 7645-7652 (2022). https://doi.org/10.1039/D2TA00697A

[S11] L. Hong, X. Wu, Y.-S. Liu, C. Yu, Y. Liu, K. Sun, C. Shen, W. Huang, Y. Zhou, J.-S. Chen, K.-X. Wang. Self-adapting and self-healing hydrogel interface with fast zn2+ transport kinetics for highly reversible zn anodes. Adv. Funct. Mater. **33**(29), 2300952 (2023). https://doi.org/https://doi.org/10.1002/adfm.202300952

[S12] B. Zhang, L. Qin, Y. Fang, Y. Chai, X. Xie, B. Lu, S. Liang, J. Zhou. Tuning zn2+ coordination tunnel by hierarchical gel electrolyte for dendrite-free zinc anode. Sci. Bull. **67**(9), 955-962 (2022). https://doi.org/https://doi.org/10.1016/j.scib.2022.01.027

[S13] N. Mittal, A. Ojanguren, D. Kundu, E. Lizundia, M. Niederberger. Bottom-up design of a green and transient zinc-ion battery with ultralong lifespan. Small **19**(7), 2206249 (2023). https://doi.org/https://doi.org/10.1002/smll.202206249

[S14] Y. Huang, J. Zhang, J. Liu, Z. Li, S. Jin, Z. Li, S. Zhang, H. Zhou. Flexible and stable quasi-solid-state zinc ion battery with conductive guar gum electrolyte. Mater Today Energy **14**, 100349 (2019). https://doi.org/https://doi.org/10.1016/j.mtener.2019.100349

[S15] S. Zhang, N. Yu, S. Zeng, S. Zhou, M. Chen, J. Di, Q. Li. An adaptive and stable bio-electrolyte for rechargeable zn-ion batteries. J. Mater. Chem. A **6**(26), 12237-12243 (2018). https://doi.org/10.1039/C8TA04298E
